# Cytarabine-Induced Bradycardia: A Case Report

**DOI:** 10.7759/cureus.30624

**Published:** 2022-10-24

**Authors:** Khalid Albsheer, Abdalla Fadul, Alaa Khalafalla, El Mustafa Abdalla, Husam Al-Dubai

**Affiliations:** 1 Internal Medicine, Hamad General Hospital, Doha, QAT; 2 Internal Medicine, Hamad Medical Corporation, Doha, QAT

**Keywords:** cardiotoxicity, chemotherapy-induced cardiotoxicity, drug-induced bradycardia, acute myeloid leukemia (aml), idarubicin, cytarabine

## Abstract

Cardiotoxicity is damage to the heart muscle, which affects its function. Chemotherapy is known to cause cardiotoxicity along with many other medications and etiologies. Many chemotherapeutic cocktails are known to be associated with cardiotoxicities, such as taxane and cisplatin. Patients might have arrhythmias, severe bradycardia, cardiomyopathy, and even cardiac arrest, so precautions are taken when such medications are started. This report presents a patient who developed severe symptomatic bradycardia after receiving idarubicin and cytarabine and was managed conservatively, along with a literature review of this entity.

## Introduction

Sinus bradycardia is when the heart rate falls below 60 beats per minute with an electrical impulse generated from the sinoatrial (SA) node with an entire conductive system [1]. It commonly occurs in elderly patients aged 65 years and above and young athletes [2]. Causes of sinus bradycardia are divided into inherent etiologies, which affect the heart primarily, such as ischemic heart disease or cardiomyopathy, and extrinsic etiologies, which mainly include drug-induced sinus bradycardia [3].

Cardiovascular complications are expected to occur in cancer patients receiving chemotherapy, and those with pre-existing cardiovascular diseases are at higher risk. Complications include arrhythmias, heart failure, pericardial disease, cardiomyopathy, and myocardial infarction.

Multiple chemotherapeutic agents were associated with cardiotoxicity, for which anthracyclines and related compounds are the most frequently observed cause. However, other agents, such as conventional cytotoxic molecularly targeted agents, were associated with cardiotoxicity [4,5]. This report includes a case of chemotherapy-induced symptomatic bradycardia as well as a literature review on the subject.

## Case presentation

**Figure 1:**
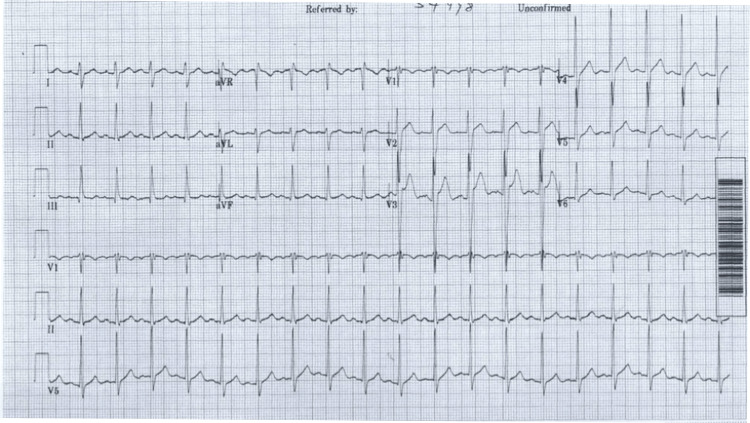
ECG on admission (baseline) This ECG was performed upon admission, and the HR was 100 beats per minute. There was no bradycardia or aberrant rhythm prior to starting cytarabine. ECG: electrocardiogram; HR: heart rate.

A 28-year-old gentleman presented to the emergency department with increasing bleeding per rectum for 10 days, associated with easy fatigability. No constitutional symptoms were present, and no other complaints were reported. No family history of malignancy was found. On presentation, he had tachycardia (Figure 1) with normal temperature (36.9°C) and blood pressure (125/75), with a pulse rate of 100 beats per minute. Physical examination was significant for small piles. Initial labs showed anemia and thrombocytopenia (Table 1). A peripheral smear revealed blastocysts. A bone marrow biopsy was done, and the patient was diagnosed with acute myeloid leukemia (AML).

**Table 1:** Basic labs Initial labs showed leukocytosis, anemia, and thrombocytopenia. Six days later, the patient developed worsening leucopenia. He also had a drop in hemoglobin and platelet count. WBC: white blood cells; HB: hemoglobin; MCV: mean cell volume.

	On admission	Six days later (febrile neutropenia)	Reference range
WBC	19	0.1	4-10 x 10^3/uL
Absolute neutrophil count	3.4	0.0	
HB	7.3	5.1	13-17 gm/dl
MCV	99.1	94.0	
Platelets	24	12	150-400 x 10^3/uL
Urea	5.2	6	
Creatinine	76	81	62-106 umol/L
Sodium	139	145	136-145 mmol/L
Potassium	4.1	3.6	3.5-5.5 mmol/L
Albumin	38	25	34-54 g/L

After reviewing the patient, the hematology team decided to start him on chemotherapy, 3 + 7 protocol (idarubicin and cytarabine). Three doses of idarubicin 18.84 mg infusion were given three days in a row, and seven doses of cytarabine 314 mg infusion were given seven days in a row.

**Figure 2:**
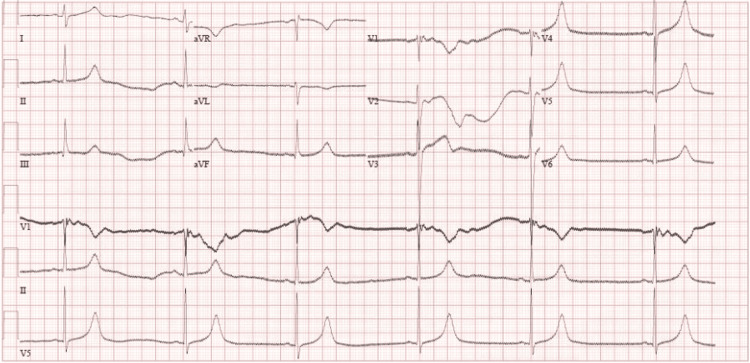
ECG showing sinus bradycardia

He had a low-grade temperature of 38.0°C. He was diagnosed with febrile neutropenia after repeated labs revealed a low neutrophil count. No source of infection was identified. He was hemodynamically stable. A sepsis workup was sent, and empirical antibiotics were started (amikacin, piperacillin-tazobactam, and caspofungin). Cultures came back negative three days later, and he became afebrile. However, he started to experience dizziness, but he had no chest pain, shortness of breath, or any other symptoms. Upon checking his vitals, he was found to have severe bradycardia with a pulse rate ranging from 30 to 40 beats per minute, blood pressure was around 153/85, and SpO2 was 100% on room air. ECG showed sinus bradycardia (Figure 2). Electrolytes, thyroid functions, and troponin T were unremarkable. Transthoracic echocardiogram showed an ejection fraction of 59% with no other abnormalities.

**Figure 3:**
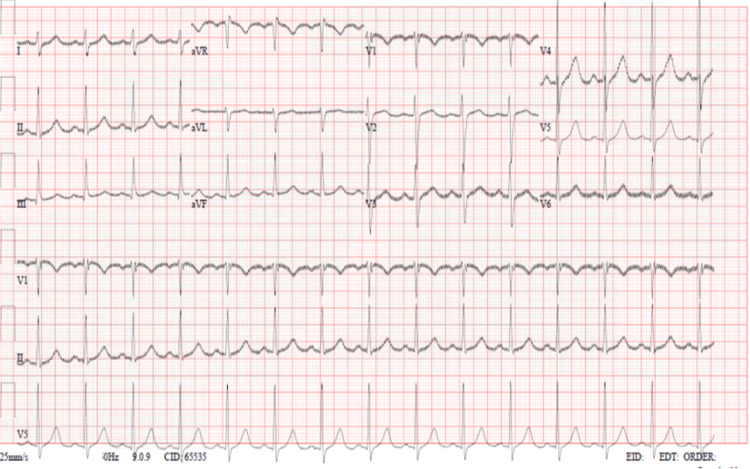
ECG three days after observation demonstrating back to baseline rhythm

The patient was transferred for observation to the coronary intensive care unit (CICU). His chemotherapy was discontinued, and over three days, his bradycardia improved (Figure 3). We believed the bradycardia was brought on by his chemotherapy treatment. He stayed under observation in CICU for two days and then he was transferred back to the hematology ward as his bradycardia was resolved and did not recur.

A week later, the patient spiked a fever and was found to have hypotension most likely due to septic shock. He was then transferred to the medical intensive care unit (MICU) and was treated with broad-spectrum antibiotics and vasopressors. The likely sources of infection were found to be a peri-anal abscess and pneumonia complicated by para-pneumonic effusion. Two days later, the patient's condition worsened. He was intubated and placed on mechanical ventilation. He then had a cardiac arrest and was declared dead after an unsuccessful cardiopulmonary resuscitation (CPR) attempt.

## Discussion

Chemotherapy-induced cardiotoxicity is a well-known entity [6]. Taxane, for example, is a pro-arrhythmic drug causing prolonged QT intervals and arrhythmias. Cisplatin is known to cause thrombosis and thromboembolic events, and anti-angiogenic therapy is known to cause hypertension and heart failure [7-10].

Bradycardia is an adverse effect of some chemotherapeutic agents. The major ones reported by the American Heart Association (AHA) are anti-microtubules like paclitaxel and thalidomide [10]. Upon searching the literature, we found that cisplatin is also reported to be associated with bradycardia [11]. Buza et al. published an article about chemotherapy and arrhythmia. In addition to the above-mentioned chemotherapeutic agents to be a cause of sinus bradycardia, they have mentioned alkylating agents like cyclophosphamide and ifosfamide, amsacrine, anthracyclines, capecitabine, and 5-fluorouracil to be the cause of sinus bradycardia [12].

In view of our patient diagnosed with AML, the “7 + 3” regimen was started. This regimen is the preferred remission induction approach in medically fit patients diagnosed with AML. It includes a seven-day continuous infusion of cytarabine and idarubicin (or daunorubicin) treatment on days one to three [13]. Although sinus bradycardia is not a well-known side effect of its use, some cases of patients who developed even symptomatic sinus bradycardia after cytarabine administration have been reported in the literature [14-16]. Idarubicin is an anthracycline antineoplastic agent known to cause cardiac toxicity leading to heart failure. ECG abnormalities observed after receiving idarubicin are mainly represented by tachycardia and arrhythmia [7].

Three days after stopping the cytarabine treatment, our patient experienced sinus bradycardia. It lasted three days before dramatically improving. Other causes of sinus bradycardia were ruled out during this time, and the patient did not take any medicine that may block the atrioventricular node and produce sinus bradycardia. Although he developed febrile neutropenia for which he received IV anti-microbial agents, he was not in severe sepsis that could explain sinus bradycardia. Additionally, anti-microbial agents administered (piperacillin-tazobactam, amikacin, caspofungin) are not known to cause sinus bradycardia; adding to this, his bradycardia improved while he was on the same antibiotics. Another hypothesis is that sinus bradycardia was caused by the combination of cytarabine and idarubicin. However, bradycardia emerged around one week after discontinuing idarubicin, making this scenario exceedingly improbable, but it cannot be fully ruled out.

## Conclusions

Chemotherapeutic drugs have been linked to sinus bradycardia. In this case, cytarabine was determined to be the most probable cause of the patient's bradycardia. The computed score was 6 on the adverse drug reaction probability scale. We believe that sinus bradycardia might be an adverse effect of cytarabine infusion. Although it is just transient, the patient should be closely monitored.
